# Biochemical Characterization of Nonamer Binding Domain of RAG1 Reveals its Thymine Preference with Respect to Length and Position

**DOI:** 10.1038/srep19091

**Published:** 2016-01-08

**Authors:** Deepthi Raveendran, Sathees C. Raghavan

**Affiliations:** 1 Department of Biochemistry, Indian Institute of Science, Bangalore, 560 012, India

## Abstract

RAG complex consisting of RAG1 and RAG2 is a site-specific endonuclease responsible for the generation of antigen receptor diversity. It cleaves recombination signal sequence (RSS), comprising of conserved heptamer and nonamer. Nonamer binding domain (NBD) of RAG1 plays a central role in the recognition of RSS. To investigate the DNA binding properties of the domain, NBD of murine RAG1 was cloned, expressed and purified. Electrophoretic mobility shift assays showed that NBD binds with high affinity to nonamer in the context of 12/23 RSS or heteroduplex DNA. NBD binding was specific to thymines when single stranded DNA containing poly A, C, G or T were used. Biolayer interferometry studies showed that poly T binding to NBD was robust and comparable to that of 12RSS. More than 23 nt was essential for NBD binding at homothymidine stretches. On a double-stranded DNA, NBD could bind to A:T stretches, but not G:C or random sequences. Although NBD is indispensable for sequence specific activity of RAGs, external supplementation of purified nonamer binding domain to NBD deleted cRAG1/cRAG2 did not restore its activity, suggesting that the overall domain architecture of RAG1 is important. Therefore, we define the sequence requirements of NBD binding to DNA.

RAGs (recombination activating genes), the nuclease responsible for V(D)J recombination consists of RAG1 and RAG2^1^,^2^. During the process of V(D)J recombination, RAG1 and RAG2 work as a heterodimer to generate a wide array of rearrangements in antigen receptors in T and B lymphocytes by rearranging V (variable), D (diversity), and J (joining) gene segments^3^. Each V, D, and J subexons are flanked by recombination signal sequence (RSS), comprising of a conserved heptamer and nonamer separated by less conserved spacer sequence of either 12 bp or 23 bp, named as 12 or 23 RSS, respectively^3^. A pair of 12 and 23 RSS is required for productive recombination, a phenomenon called as 12/23 rule^4^. The whole process of V(D)J recombination can be divided into two phases, DNA cleavage and joining. During the cleavage phase, RAGs recognize and bind to RSS present at the edge of each of the V, D and J sub exons and induce a nick at 5′ end of heptamer^5^. The resulting 3′OH group attacks the opposite strand by transesterification reaction, resulting in hairpin formation, which upon cleavage by DNA-PKcs and artemis culminates into a double-strand break (DSB)^6^. The resulting coding ends and blunt signal ends are further processed and joined by nonhomologous DNA end joining (NHEJ)^7^.

Previous studies have shown that, besides the recombination signal sequences present at antigen receptor loci, there are several cryptic RSSs present elsewhere in the genome^8^. It has been observed that RAGs can misrecognize and cleave such cryptic RSS leading to chromosomal translocations in leukaemia and lymphoma^9^,^10^,^11^,^12^.

Besides its role in V(D)J recombination as a sequence-specific nuclease, RAGs can act as a structure-specific nuclease wherein it recognizes altered DNA structures and introduces nick at double strand (ds)-single strand (ss) transition. Studies have shown that RAGs can cleave hairpins, heterologous loops, symmetrical bubbles, triplex, G-quadruplex etc^13^,^14^,^15^,^16^,^17^,^18^. Recent studies have revealed that the structure-specific activity of RAGs is regulated by sequence of both the single-stranded DNA and flanking double-stranded DNA^19^,^20^. Further, the RAG cleavage efficiency at heteroduplex DNA was enhanced when a nonamer was present adjacent to it^21^.

Full length RAG1 consists of N-terminal domain, nonamer binding domain (NBD), central domain (CD) and C-terminal domain (CTD). However, a truncated version of RAG1, called core RAG1 (cRAG1) harbours all the domains (NBD, CD and CTD) essential for its catalytic activity *in vitro* (Fig. 1A)^22^,^23^. The core RAG complex has been crystallized recently^24^. NBD at the N-terminus of cRAG1, recognizes and binds specifically to nonamer of RSS and thus aids in anchoring RAGs onto RSS, while other domains are responsible for its catalytic activity and its interaction with RAG2^25^,^26^. NBD of RAG1 has been crystalized in complex with a 14-mer duplex DNA containing nonamer sequence^27^. The study revealed that NBD exists as a homodimer in solution and interacts with two molecules of DNA, each NBD monomer interacting with both DNA molecules.

The flexibility of RAG proteins to recognize and bind to cryptic RSS questions the specificity of NBD of RAG1 in recognizing and binding to nonamer alone and its role in pathological function of RAGs. Therefore, evaluation of the biochemical interactions and substrate preference for nonamer binding domain of RAG1 is important in understanding its role in genomic instability and cancer. Since a nonamer adjacent to a non-B DNA structure could facilitate its cleavage, characterizing the role of NBD in structure-specific activity of RAGs is also of significance. Thus, in the present study we characterize the binding properties of NBD, which was overexpressed and purified from bacteria. We report specific binding of NBD to both 12 and 23 RSS using a biochemical system. However, NBD did not bind to heteroduplex DNA, irrespective of the sequence of the single-stranded region. Interestingly, when a nonamer was present next to a heteroduplex DNA, NBD exhibited robust binding. NBD binding was specific to thymines with Kd value of 0.4 μM when single-stranded DNA containing poly A, C, G, T were used. A minimum length of 23 nt was essential for NBD binding to homothymidine stretches. On a double-stranded DNA, NBD could bind to A:T stretches, but not to G:C stretches or random sequences. Therefore, we define the sequence requirements of NBD binding to double- and single-stranded DNA.

## Results

### Overexpression and purification of NBD of RAG1

**Figure 1:**
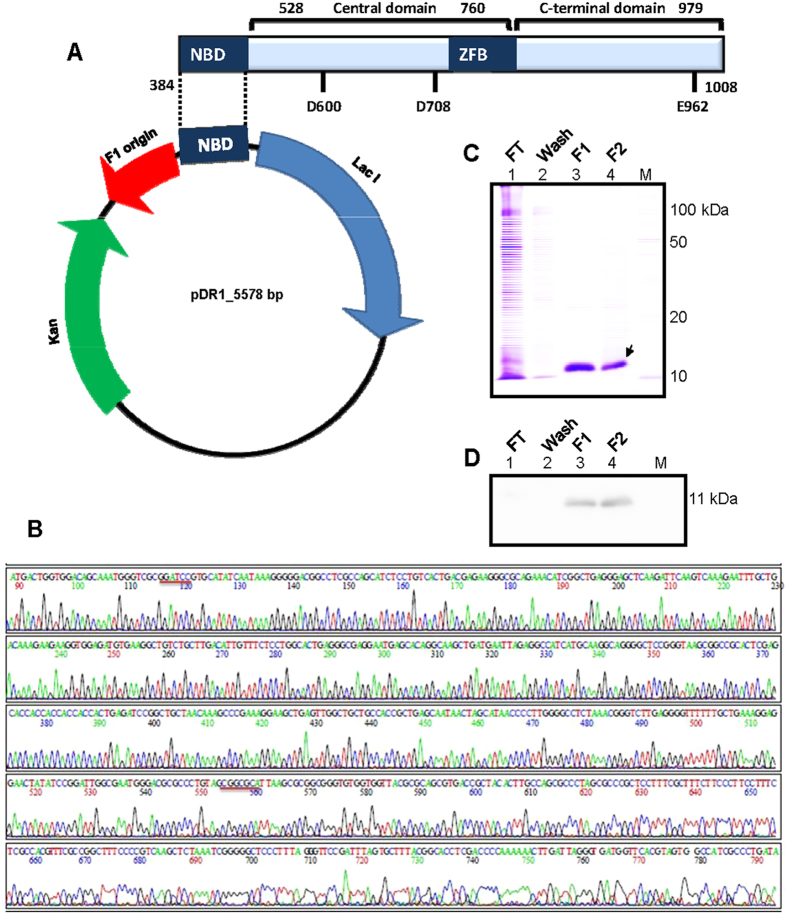
Cloning and purification of nonamer binding domain of RAG1. (**A**) The nonamer binding domain of murine RAG1, corresponding to amino acids 384–461 was cloned into pET28(a). The vector map of resulting pDR1 with major components indicated is presented. The domain architecture of cRAG1 with domains marked is also indicated. (**B**) The electropherogram of the cloned DNA sequence confirming NBD. The restriction sites spanning the insert are underlined. (**C**,**D**) The NBD was overexpressed in *E. coli* BL21 and purified through Ni-NTA affinity chromatography. Purity of the protein was tested on a SDS PAGE stained with CBB (**C**) and identity was confirmed by western blotting (**D**). ‘M’ is molecular weight ladder. ‘FT’ is flow through; ‘F1’ and ‘F2’ are two independent fractions.

The nonamer binding region corresponding to amino acids 384–461 were PCR amplified from *RAG1* gene. The amplified product was cloned into bacterial expression vector, pET28(a), resulting in pDR1 (Fig. 1A). Following confirmation of NBD by DNA sequencing (Fig. 1B), it was used for transformation of *E. coli* BL21. NBD was overexpressed and purified from bacteria by virtue of its N-terminus His tag (hexa histidine) using Ni-NTA affinity chromatography. Purity of the eluted fractions was confirmed by SDS-PAGE (Fig. 1C). The band due to NBD showed a mobility at the expected molecular weight of 11 kDa. The identity of the protein was further confirmed by immunoblotting (Fig. 1D).

### NBD binds to nonamer in the context of RSS and heteroduplex DNA

**Figure 2:**
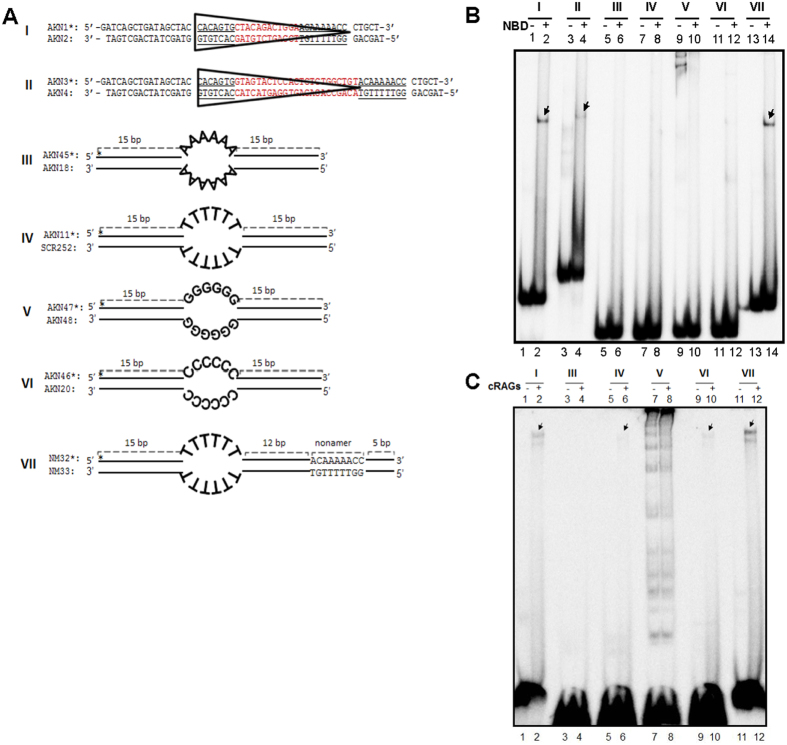
Comparison of binding potential of NBD with different RAG target sequences. (**A**) Diagrammatic representation of oligomeric DNA substrates used for the study. Substrate I harbours a 12RSS, while II is a 23RSS. In both cases, the heptamer and nonamer are underlined. Substrates III to VI are heteroduplex DNA containing either six nucleotides of adenine, cytosine, guanine or thymine at single-stranded region as indicated. Substrate VII is a heterduplex DNA in which thymine bubble is separated from a nonamer by 12 bp. (**B**) Polyacrylamide gel profile showing comparison of NBD binding to RSS and different heteroduplex DNA substrates. Following NBD binding (100 ng), the products were resolved on a 4% native polyacrylamide gel. The bands due to NBD binding are indicated. (**C**). Polyacrylamide gel profile showing cRAG binding to 12RSS and different heteroduplex DNA substrates. The bands due to protein-DNA complex are indicated with arrows. Note different levels of intermolecular G-quadruplex formation in oligomer V due to presence of potassium glutamate in the reaction buffer.

In order to test whether the purified NBD could bind to its physiological substrates, recombination signal sequences, gel mobility shift assays were performed. Oligomers harbouring 12/23 RSS were synthesized, purified, radiolabelled and annealed to make double-stranded DNA substrates (Fig. 2A). The substrates were subjected to NBD binding (100 ng) assay and the products were resolved on a 4% native polyacrylamide gel. Results showed distinct binding of NBD to 12 and 23 RSS resulting in a retarded mobility of oligomers, which is indicated by arrows (Fig. 2B, lanes 1–4).

Previously it has been shown that RAG complex can cleave heteroduplex DNA in a sequence dependent manner^19^. Based on the above results, we tested whether NBD can bind to heteroduplex DNA. Oligomers containing heteroduplex DNA with adenine, thymine, guanine or cytosine at single stranded region were synthesized (Fig. 2A). EMSA studies with NBD and heteroduplex DNA did not show detectable binding irrespective of the sequence of the single-stranded region (Fig. 2B, lanes 5–12). Interestingly, NBD binds to the heteroduplex DNA when a nonamer was present adjacent to it (Fig. 2B, lanes 13–14). Thus, NBD can bind to nonamer irrespective of whether it is positioned adjacent to a heptamer or heteroduplex DNA. The same was also verified using NBD purified from an independent expression vector described previously^27^ (data not shown).

In order to assess the specificity of the observed NBD binding with RSS, a competition experiment was performed. NBD (~200 ng) was incubated with 4 nM of labelled DNA bearing a consensus 12RSS (I), to which increasing amount of either unlabelled 12RSS or a non-specific oligonucleotide lacking both nonamer and heptamer (XIV) was added (Fig. S1A,B). Results showed concentration dependent decrease of NBD binding to radiolabelled RSS, with an increase in the concentration of unlabelled 12RSS (Fig. S1A,B). However, such a concentration dependent decrease was not evident when competition experiment was carried out with non-specific DNA (Fig. S1C,D). Even 7-fold excess of nonspecific competitor did not result in any significant decrease of the shifted complex (Fig. S1D). Thus, our results show that the binding of NBD is specific to the nonamer of RSS and that it cannot bind to random double-stranded oligomers when nonamer is not present.

Based on the observed NBD binding preference above, we were interested in evaluating binding preference of cRAGs to RSS and heteroduplex DNA. In order to test this, purified cRAG1/cRAG2 complex (200 ng) was incubated with different hetroduplex DNA, heteroduplex DNA with a flanking nonamer and 12RSS. Results showed distinct protein DNA complex with heteroduplex DNA, when cytosines or thymines were present at single-stranded DNA (Fig. 2C, lanes 5, 6, 9, 10). RAG binding was also observed when heteroduplex DNA harbouring nonamer was used (Fig. 2C, lanes 11,12). Besides, a band due to cRAG binding to 12RSS was also observed. However, we did not observe any detectable binding of cRAGs to heteroduplex DNA bearing A and G sequences at single-stranded region (Fig. 2C). These results were also consistent with previous studies where RAG cleavage on heteroduplex DNA was dependent on DNA sequence as well as presence of a nonamer^19^,^21^.

### NBD binding is dependent on presence of a nonamer of V(D)J recombination

**Figure 3:**
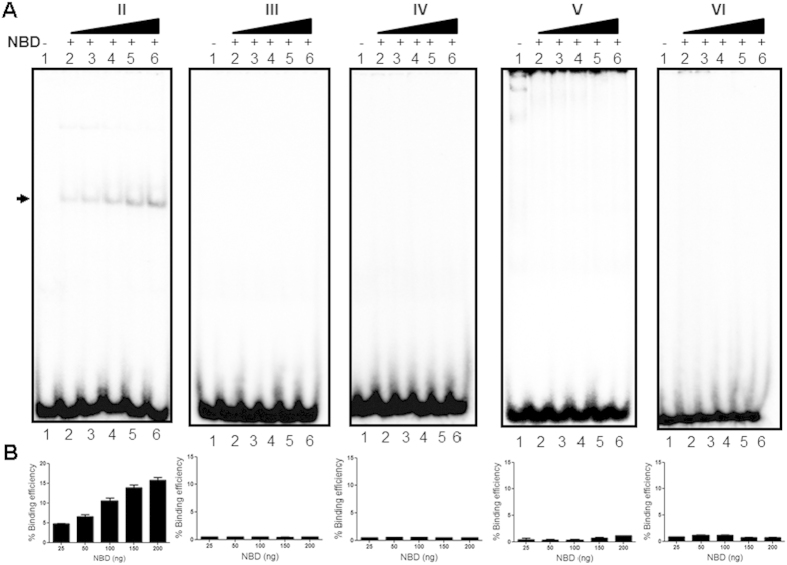
Evaluation of NBD binding to RSS and heteroduplex DNA at increasing concentrations of proteins. (**A**) Polyacrylamide gel profile showing NBD binding to 23RSS and different heteroduplex DNA substrates, at increasing levels of NBD (25, 50 100, 150 and 200 ng). Following NBD binding, the products were resolved on a 4% native polyacrylamide gel and band due to RAG binding is indicated. (**B**) Bar diagram showing quantification of the NBD binding on 23RSS and heteroduplex DNA structures at varying protein concentrations.

Protein titration experiments showed no detectable binding of NBD to different heteroduplex DNA, even when increasing concentrations (25, 50, 100, 150, 200 ng) of purified NBD was used (Fig. 3A,B). In contrast, when increasing concentrations of NBD was incubated with 23RSS, a concentration dependent increase in binding was observed with a binding constant of 13.9 +/− 3.3 μM (Fig. 3). These results suggest that presence of a RSS is important for binding of NBD. These results were also consistent with previous studies wherein structure-specific activity of RAGs was unaffected when NBD was deleted from RAG1^28^

Since above results suggest that NBD bind to a heteroduplex DNA, when a nonamer is present flanking to it, we were interested in testing the binding was indeed due to presence of nonamer. To examine this, oligomeric DNA with conventional nonamer was replaced with a scrambled nonamer and used for binding studies (Fig. S2A). Results showed no significant binding with the heteroduplex DNA adjacent to scrambled nonamer as compared to conventional nonamer (Fig. S2B). Thus, observed NBD binding can be attributed to presence of a nonamer of V(D)J recombination.

### NBD binds to single-stranded and double-stranded DNA when thymines are present

**Figure 4:**
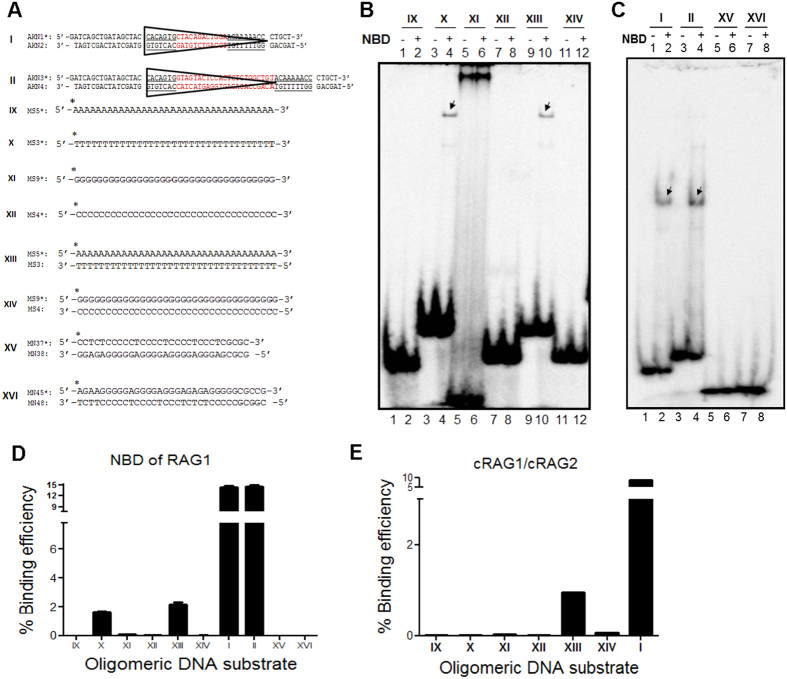
Evaluation of affinity of NBD to thymidine containing single- or double-stranded DNA compared to other nucleotides. (**A**) Diagrammatic representation of oligomeric DNA substrates used for the study. Substrates IX to XII are single stranded DNA containing either poly A, T, G or C sequences. Substrate XIII and XIV are dsDNA containing polynucleotides of A/T and G/C, respectively. Substrates XV and XVI are ds DNA containing random nucleotides (lacks A/T stretches). (**B,C**) Polyacrylamide gel profile showing NBD binding to different ss and ds DNA substrates. Following NBD binding the products were resolved on a native PAGE as described above. The bands due to NBD binding are indicated. (**D**) Bar diagram showing quantification of NBD binding to ss and ds DNA substrates. (**E**) Bar diagram showing quantification of cRAGs binding profile to ss and ds DNA substrates.

To evaluate the binding preferences of the NBD towards single-stranded DNA, oligomers containing continuous stretches of adenine, guanine, thymine or cytosine were synthesized (Fig. 4A. IX-XII). The radiolabelled oligomers were incubated with ~200 ng of NBD (15 min, 30 °C) in binding buffer and the products were resolved on a 4% native polyacrylamide gel. Results showed specific binding of NBD to oligomer containing poly T, while binding was not observed in case of ss poly A, G and C substrates (Fig. 4B, lanes 1–8; 4D). When double-stranded DNA containing A/T or G/C sequences were used for binding studies, NBD binding was observed in the case of double-stranded DNA with A/T sequences (Fig. 4B, lanes 9, 10). However, we could not find any binding when ds DNA containing G/C sequence was used (Fig. 4B, lanes 11, 12). To further evaluate the binding properties of NBD, the protein was incubated with double-stranded oligomers containing random sequences (Fig. 4A. XV, XVI). Results did not show any detectable binding when nonamer was absent (Fig. 4C, lanes 5–8; 4D). Oligomers bearing consensus 12 and 23RSS served as positive control for binding. Thus our results show that NBD binds preferentially to thymine sequences on a single-stranded DNA, and T/A sequences in ds DNA. This also suggests that binding of NBD to nonamer of RSS is by virtue of the A/T stretch as part of the nonamer.

Based on above observation, we were inquisitive to see if cRAGs show such sequence preferences, when it bind to sequences apart from nonamer of RSS. cRAGs were incubated (200 ng) with different ss and ds DNA and the complex was resolved on 4% native polyacrylamide gel. Results did not show any significant binding in case of ss poly A, T, G and C DNA (Fig. S3). Interestingly, a weak, but distinct protein-DNA complex was observed when cRAGs were incubated with polynucleotide ds DNA with A/T was used; however such a binding was absent when ds DNA with G/C sequences were used for binding. Since observed A/T binding by cRAGs was much less compared to 12RSS (Fig. 4E), it is clear that the overall architecture of the protein is important to help in achieving the specificity of RAGs. However, these observations will have pathological implications and will help in explaining RAG mediated genomic instability.

### BLI studies confirm the thymidine specificity of NBD at single molecule level

**Figure 5:**
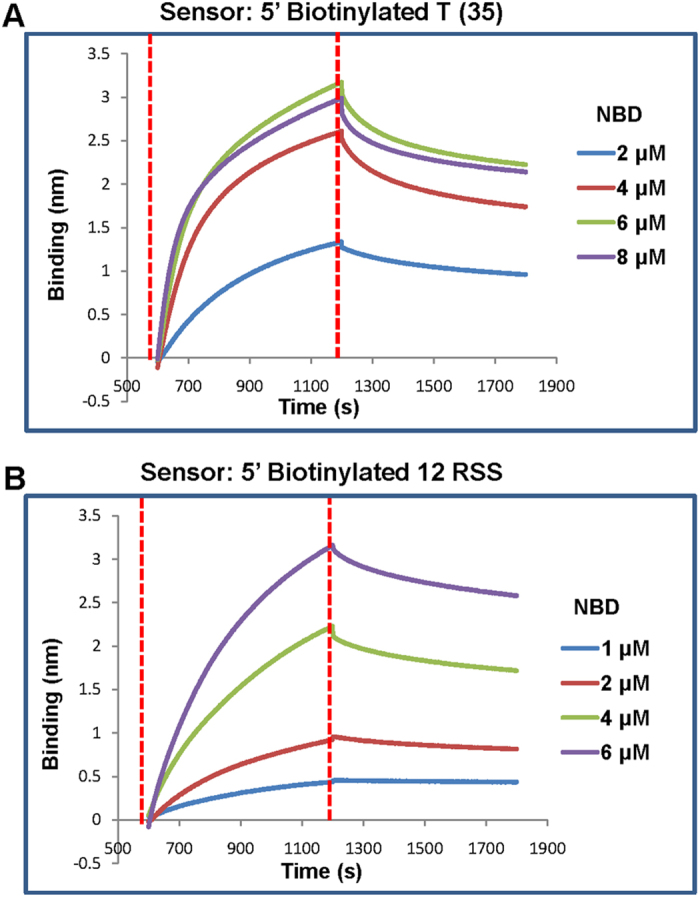
Biolayer interferometry studies showing comparison of efficiency of NBD interaction to poly thymidine with that of a DNA comprising 12RSS. (**A**) Biolayer interferometry sensorograms depicting the real time binding of NBD protein (2, 4, 6 and 8 μM) with a 5′ biotinylated poly T (35 nt) DNA. The red dotted lines indicate start of binding (left) and dissociation (right). (**B**) BLI sensorograms depicting the real time binding of a 5′biotinylated 12RSS biosensors to NBD protein (1, 2, 4 and 6 μM). The red dotted lines indicate start of binding (left) and dissociation (right). The real time binding curves were used to compute equilibrium dissociation constant by globally fitting the rate equation for 1:1 kinetics to the data.

In order to test specificity of NBD binding to poly T substrates at single molecule level, Biolayer interferometry (BLI) experiments were performed^29^. For this, 5′-biotinylated DNA harbouring poly T (35 nt) or 12 RSS (50 bp) was synthesized and resuspended in 1× PBS. Biotinylated DNA was immobilized to streptavidin sensor surface, following which increasing concentrations of purified NBD protein were allowed to interact. The binding of protein to the biosensor increases the thickness of the layer on sensor tip surface, which in turn is measured by BLI as an interference wavelength shift. To measure binding kinetics of NBD to DNA with poly T or 12RSS, the sensors immobilized with 5′ biotinylated DNA were transferred to wells containing assay buffer and then to the wells containing assay buffer with NBD protein to measure the interaction. Results showed a significant shift in the association curve upon addition of NBD (2, 4, 6, 8 μM) in a concentration dependent manner (Fig. 5A). These results confirm the ability of NBD to interact with poly T DNA, further emphasizing NBD binding preference towards thymines. Efficient binding was also observed when BLI experiment was performed using 12RSS and NBD (1, 2, 4, 6 μM) (Fig. 5B).

The real time binding curves generated were fit globally to a 1:1 binding model and equilibrium dissociation constant (Kd) was calculated. Interestingly, results showed a robust binding of NBD to poly thymidine DNA with a Kd value of 0.45 ± 0.16 μM (mean ± SD), which was less than that of 12RSS (4.02 ± 2.6 μM), emphasising the fact that NBD can bind to poly T DNA. >22 thymines are required for NBD binding to poly thymine containing ss DNA.

**Figure 6:**
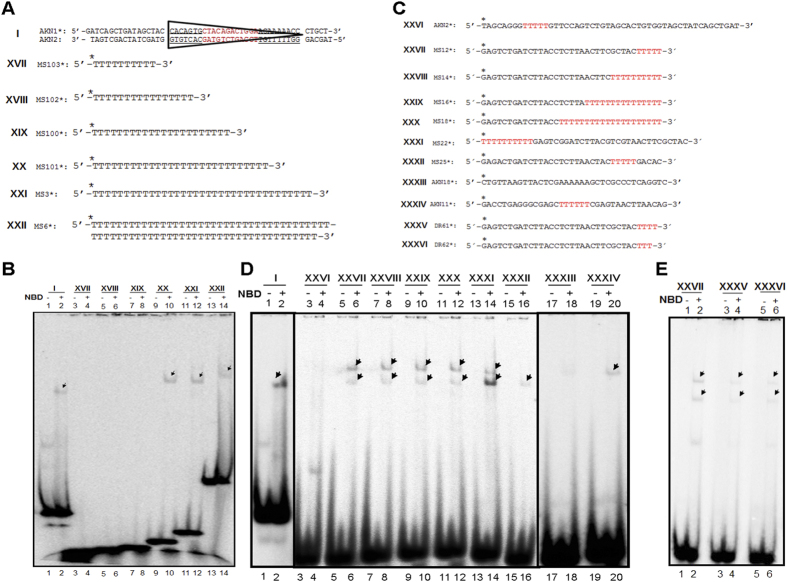
Evaluation of minimum length and position of thymines required for binding of NBD. (**A**) Diagrammatic representation of oligomeric DNA substrates containing poly T (10, 16, 22, 28, 35, 75 nt) used for the study. 12RSS is also depicted. (**B**) Polyacrylamide gel profile showing NBD (200 ng) binding to DNA substrates with different length poly T stretches. The bands due to NBD binding are indicated. (**C**). Diagrammatic representation of oligomeric DNA substrates containing stretches of T (highlighted in red) used for the study. Substrate XXVII to XXX harbour 5, 10, 15, 20 thymines at the 3′ end. Substrate XXXI harbours 10 thymines at the 5′end. Substrate XXVI and XXXII possess stretch of thymine, but not towards the ends while XXXIII lacks thymine stretch. Substrate XXXIV has a stretch of 6 thymines in the centre. Substrates XXXV, XXXVI have 4 and 3 thymines at the 3′ end. (**D**) Polyacrylamide gel profile showing NBD binding to DNA substrates with different length poly T stretches. The bands due to NBD binding are indicated. (**E**) Polyacrylamide gel profile showing NBD binding to DNA containing 4 and 3 thymines at the 3′end (XXXV, XXXVI). DNA with 5 thymines at the 3′end served as positive control (XXVII).

Above results showed that although NBD binds to the single-stranded poly T DNA, it failed to do so when a short single stranded poly T stretch was present as part of a heteroduplex DNA. Hence, we further investigated NBD binding to poly T substrates to determine the minimum length of thymines required for NBD binding. Oligomers with number of thymines ranging from 10–75 nt (10, 16, 22, 28, 35 and 75 nt) were synthesized, radiolabelled and subjected to NBD binding experiments (Fig. 6A). Results showed efficient binding of NBD with the poly thymine containing ssDNA when the length was 28, 35 or 75 nt (Fig. 6B, lanes 9–14). However, when 10, 16 and 22 nt length poly T containing DNA was used, we did not find any detectable NBD binding (Fig. 6B, lanes 3–8). 12RSS served as the positive control for NBD binding (Fig. 6B, lanes 1, 2). Thus, our results suggest that >22 nt of poly T stretches may be needed for efficient binding of NBD in the context of single stranded DNA.

### A stretch of 5 thymines is sufficient for definitive NBD binding to ss DNA

Further to evaluate if the position of the thymines play a role in NBD binding to poly thymines containing ss DNA, oligomers with varying length of thymines at 5′ or 3′ terminus were synthesized (Fig. 6C). We find that efficient NBD binding was evident even when 5 thymines were present at the 3′ end and there was not much difference in the NBD binding between stretch of 5 and 20 thymines (Fig. 6D, lanes 5–12). We also observed efficient binding of NBD when the thymines were positioned at the 5′ end (Fig. 6D, lanes 13, 14). With the decrease in length of thymines at the 3′ from 5 nucleotides to 4 and 3, resulted in significantly reduced NBD binding intensity (Fig. 6E, 3–6). Besides, binding was weak when the thymine stretch was interspaced (Fig. 6D, lanes 15, 16, 19, 20). Importantly, there was no detectable binding when thymine stretches were absent (Fig. 6D, lanes 17 and 18). Moreover, NBD binding was undetectable even when 5 thymines were present on a single stranded DNA of 50 nt length (Fig. 6D, lanes 3 and 4), indicating both overall length of the DNA and number of thymine can play a role in determining affinity of NBD to single stranded DNA (Fig. S4). In most cases of NBD binding to ss DNA with thymines, we observed two shifted bands. This could be due to difference in the binding stoichiometry and cooperativity, which is dependent on the favourable conditions. Thus, our study reveals that both length and position of thymines play an interesting role in governing NBD binding preference to different DNA substrates in a context dependent manner on single-stranded DNA.

**Figure 7:**
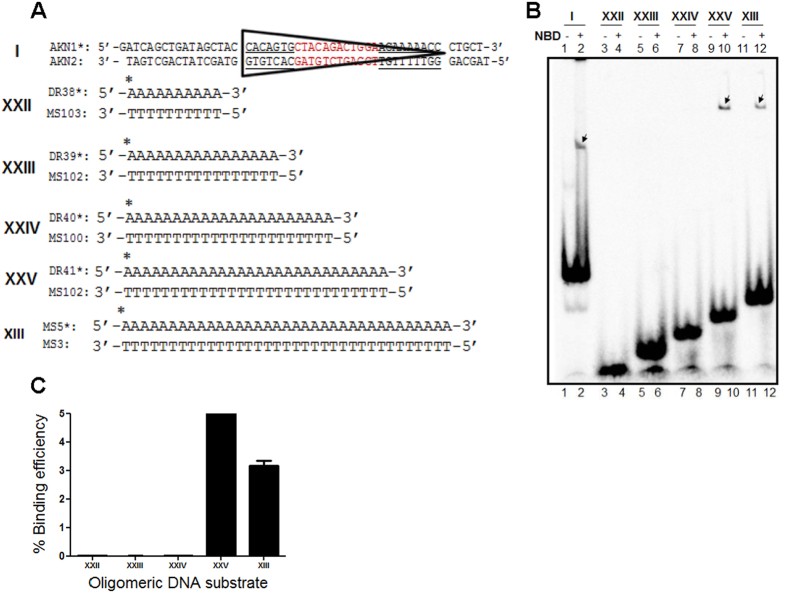
Evaluation of minimum length of double-stranded A:T stretch required for efficient binding of NBD. (**A**) Diagrammatic representation of oligomeric DNA substrates containing AT stretch (10, 16, 22, 28, 35 bp) used for the study. (**B**) Polyacrylamide gel profile showing NBD binding to DNA substrates with different length poly A:T stretches. The bands due to NBD binding are indicated. (**C**) Bar diagram showing quantification of NBD binding to different A:T DNA.

We have also investigated the minimum length of poly A/T sequence required for NBD binding. Oligomers with A/T sequences ranging from 10 to 35 bp (10, 16, 22, 28 and 35 bp) were synthesized, radiolabelled and subjected to NBD binding assay (Fig. 7A). Results showed efficient binding of NBD when the length of poly A/T sequence was 28 and 35 bp (Fig. 7B, C lanes 9–12). However, no detectable binding was observed when other poly A/T DNA substrates were used (Fig. 7B, C lanes 3–8). 12RSS served as the positive control for NBD binding (Fig. 7B, lanes 1, 2). Thus, our results suggest that NBD binding to poly T or poly A/T stretch in the genome may be restricted by the minimal length requirement. This might reduce the nonspecific RAG binding in the genomic context.

### External addition of purified NBD could not retrieve function of NBD-deleted core RAGs

**Figure 8:**
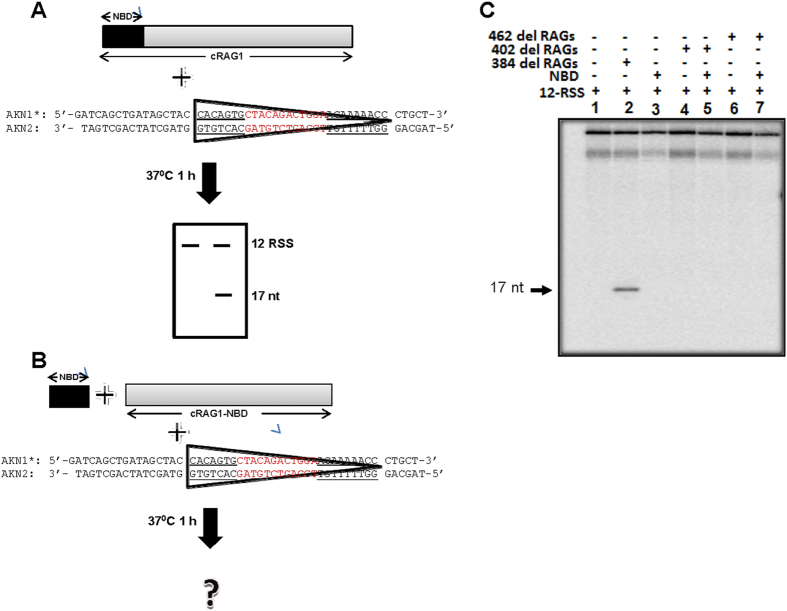
Reconstitution of sequence-specific activity of cRAG1 deficient in NBD with externally supplemented purified NBD. (**A**) Schematic representation of steps involved during reconstitution assay for RAG nicking activity on a 12 RSS. Schematic showing cRAG1 represented by a rectangular box, of which region indicated in black is NBD domain. The protein was incubated with γ-^32^P labelled 12RSS at 37 °C and resolved on denaturing PAGE to visualize the cleavage products. (**B**) Schematic presentation of cRAG1 following truncation of NBD domain. Reconstitution was performed by external addition of the purified NBD protein as indicated. The reconstituted protein complex was incubated with γ-^32^P labelled 12RSS at 37 °C and resolved on denaturing PAGE to assay the nicking activity. (**C**) Polyacrylamide gel profile showing reaction products obtained following RAG cleavage on 12RSS, using a NBD deleted RAG1 complex ([402/462–1040 RAG1]/cRAG2), in the presence of externally added purified NBD protein.

Since it has been shown that NBD deleted cRAG1/cRAG2 could not cleave RSS^28^, we were interested in examining, whether purified nonamer binding domain could help NBD deleted cRAG1/cRAG2 complex to regain the sequence specific activity. In order to evaluate this, NBD deleted cRAG1 (Δ462 and Δ402) were co-expressed with cRAG2 and purified from human kidney epithelial cells^28^. *In vitro* RSS cleavage assay was carried out on 12RSS by incubating NBD deleted cRAG1/cRAG2 complex with purified NBD protein and the products were resolved on a denaturing polyacrylamide gel (Fig. 8A). The γ-^32^P label at the 5′ end of the substrate allows identification of the nicked product after analysis by denaturing gel electrophoresis. MBP cRAG1/cRAG2 acted as positive control, while DNA substrate alone and substrate incubated with NBD served as negative control. Results showed a specific band at 17 nt position due to RAG cleavage when MBP cRAGs were used for the assay (Fig. 8C, lanes 1, 2). Further, NBD alone (Fig. 8B,C, lane 3) or either of the NBD deleted version of the RAG1/RAG2 complex (Fig. 8C, lane 4–6) failed to cleave at the RSS, which was consistent with previous studies^28^. However, even after adding the purified NBD to cRAG1 lacking the NBD/cRAG2 did not result in any cleavage, suggesting that external addition of NBD could not reconstitute the RAG cleavage action (Fig. 8C, lane 5, 7). These results suggest that the domain organisation of RAG1 would play an important role during its function.

## Discussion

In the present study, we characterize the binding properties of nonamer binding domain of RAG1. We find that purified NBD can bind to the nonamer irrespective of whether it is positioned adjacent to a heptamer of V(D)J recombination or a hetroduplex DNA. Further, the binding of NBD was specific to double-stranded DNA with RSS, while it showed no binding towards ds DNA containing random sequences when nonamer was absent. Thus our study establishes that NBD binds to the nonamer sequence of RSS, which is in line with previous reports, that suggested NBD as the domain responsible for binding to recombination signal sequences during V(D)J recombination^25^,^26^. Previous studies have also provided evidence for role of nonamers during cryptic signal cleavage by RAGs, during chromosomal rearrangements involved in leukaemia, wherein RAGs bind to a nonamer and cleaved at a CAC site rather than a consensus heptamer^9^,^30^.

Interestingly, it was noted that NBD preferentially binds to poly T, when it was present on a single-stranded DNA. It did not show any binding towards poly G, poly A or poly C. In addition, a minimum of 5 nt stretch of thymine was required for NBD binding when it was present in a single-stranded DNA with random sequence with an overall length of 35 nt. Double stranded DNA containing A/T stretches were preferred over G/C sequence during NBD binding. These results suggest that NBD interacts with thymines on an A/T rich sequence, providing mechanistic basis for its interaction with a nonamer in a RSS. Besides, the observed A/T binding preference over GC indicates the potential of RAG binding to A/T rich fragile regions^31^,^32^. Binding and cleavage on such regions by RAGs may explain genomic instability associated with those repeat sequences. This may also provide relevance for the observed RAG expression in nonlymphoid tissues^33^,^34^ which needs to be studied further.

The binding preference of purified NBD for ss poly T and ds A/T stretch also indicates that, binding of NBD is not restricted to nonamer of RSS, rather a stretch of A/T sequence may become target for RAGs. A length of >22 nt of poly T was required for NBD binding when single strand homopolymer was used, while for ds A/T sequence, the minimum required length was >28 bp. This observation might be difficult to reconcile, considering the fact that a 9 nt nonamer is sufficient for RAG binding on a 12 and 23RSS. It is possible that a short stretch of T or A/T region such as 5–10 nt may be enough for initial anchoring of RAGs, however, a longer length of DNA may be needed for whole protein to bind and cleave the DNA. Our observation that a minimum of 5 nt of thymine was sufficient for NBD binding on a 35 mer oligomer was indeed consistent with such a conclusion.

Besides, position of the thymines has minimal role with respect to NBD binding but has an impact on its binding efficiency. Therefore, our observations in conjunction with past studies suggest that the affinity of NBD to nonamer of RSS is by virtue of the A/T stretch as part of the nonamer^25^,^26^,^27^. Further, external addition of NBD to NBD deleted cRAG1/cRAG2 did not help regain its sequence-specific cleavage activity (Fig. 8), indicating that domain organisation plays a crucial role in the function of RAG1 as described recently^24^.

Although it has been reported that NBD binds specifically to nonamer of RSS, its specificity during RAG cleavage at non-B DNA structure is not well understood^18^,^21^. It has been shown that, deletion of nonamer binding region did not make any difference in structure-specific cleavage activity of RAGs^28^,^35^. Studies have also shown that different domains of RAG1 have specific roles in V(D)J recombination at single-stranded DNA^36^,^37^. Although NBD of RAG1 is not essential for the structure-specific activity of RAGs, the enhancement in the cleavage at the altered DNA structures in presence of an adjacent nonamer implies that NBD could potentiate the cleavage at altered DNA structures when a nonamer is present in its close vicinity^21^.

In a recent study, NBD of RAG1 was crystallized and results showed that GGRPR motif of NBD interacts with thymines in the nonamer^27^. The thymidine preference observed by us in our biochemical study is in accordance with the previous observations and provide importance of thymines in recognition and binding by NBD of RAG1. The preference for the AT stretch by NBD might play a crucial role in RAG mediated misrecognition and cleavage, leading to lymphoma and leukaemia^38^. Further, in a recent study it has been shown that mutation to NBD of RAG1 reduced the overall number of binding events observed even at regions lacking nonamer. This falls in line with our observation of NBD preference to sequences apart from nonamer^39^. Although as a topologically independent domain, NBD showed preference to AT rich sequences, whether the observation holds true in the context of genome as well as physiological scenario needs to be investigated.

## Methods

### Enzymes, chemicals, and reagents

Chemicals and reagents used in the study were purchased from Sigma Chemical Co. (St. Louis, MO), Amresco (USA) and SRL (India). DNA-modifying enzymes were obtained from New England Biolabs (Beverly, MA) and Fermentas (Glen Burnie, MD). Culture media were from Sera Laboratory International Limited (West Sussex, UK), and fetal bovine serum (FBS) and PenStrep from Invitrogen (USA). Antibodies were purchased from Santa Cruz Biotechnology (USA) and radioisotope-labelled nucleotides from BRIT (Hyderabad, India).

### Oligomers

Oligomers were synthesized by Sigma Aldrich, India and IDT, USA. The sequences of the oligomers used in the study are presented (Table S1). The oligomers were purified using 8–15% denaturing PAGE and when required radiolabelled as described below. In order to prepare double-stranded DNA, radiolabelled oligomer was annealed with its unlabelled complementary strand (five-fold excess) in 100 mM NaCl and 1 mM EDTA in boiling water for 10 min followed by gradual cooling as described earlier^40^.

### 5′ end-labelling of oligomers

The 5′ end-labelling of the oligomeric DNA was performed using T4 polynucleotide kinase and [γ-^32^P] ATP at 37 °C for 1 h, as described^17^. The labelled substrates were purified using Sephadex G-25 column and stored at −20 °C until use.

### Preparation of oligonucleotide substrates

The oligomeric DNA containing 12RSS was created by annealing γ-^32^P labelled AKN1 with unlabelled complementary oligomer AKN2. Radiolabelled AKN3 was annealed to unlabelled AKN4 to obtain double-stranded DNA containing 23RSS. AKN11/SCR252, AKN18/AKN45, AKN47/AKN48 and AKN46/AKN20 were annealed to obtain substrates with 6 nt bubbles of sequence TTTTTT/TTTTTT, AAAAAA/AAAAAA, GGGGGG/GGGGGG and CCCCCC/CCCCCC, respectively^19^. Substrate harbouring (T/T)6 bubble and nonamer downstream to it by 12 nt was made by annealing labelled NM32 to unlabelled NM33^21^, while the substrate harbouring (T/T)6 bubble with scrambled nonamer was made by annealing labelled DR59 to unlabelled DR60. Oligomers without A:T stretch were obtained by annealing labelled MN37 and MN45 with unlabelled MN38 and MN46, respectively^41^. Oligomers with A:T stretch were obtained by annealing substrates MS103/DR38, MS102/DR39, MS100/DR40, MS101/DR41 and MS3/MS5, respectively. Oligomer with G:C stretch was obtained by annealing labelled MS9 to unlabelled MS4.

The DNA substrates harbouring 3, 4, 5, 10, 15 and 20 thymines at the 3′ end were obtained by labelling DR62, DR61, MS12, MS14, MS16 and MS18, respectively. Substrate harbouring 10 thymines at the 5′ end was generated by labelling MS22. Substrate lacking thymine stretch was obtained by labelling AKN2 and substrate lacking thymine stretch at 3′ or 5′ ends was obtained by labelling MS25. Substrate with a stretch of 6 thymines in the centre was obtained by labelling AKN11.

### Cloning of nonamer binding doamin (NBD) of RAG1

Oligomers, AKN150 and AKN139 were used for amplifying NBD (DNA corresponding amino acids 384–461) of mouse RAG1. Following BamHI and NotI double digestion, the amplified product was cloned into bacterial expression vector pET28(a), resulting in the plasmid pDR1. The clones were checked by restriction digestion and were confirmed by DNA sequencing (SciGenom Cochin, India).

### Expression and purification of NBD

NBD was overexpressed by transforming *E. coli* BL21 with pDR1 [A(600) - 0.5, followed by 1 mM IPTG induction] and growing for 16 h at 16 °C. Cells were lysed in protein extraction buffer (20 mM sodium phosphate buffer, 500 mM NaCl, 2 mM beta mercaptoethanol, 1% triton X-100, 10% glycerol) with 10 mg/ml lysozyme, followed by sonication. The soluble fraction of the cell lysate was purified using Ni-NTA affinity chromatography as described earlier^42^. Protein was eluted with increasing concentration of imidazole and loaded on a 12% SDS PAGE to determine the purity.

### Expression and purification of core RAGs and its mutants

cRAG1 (core RAG1, amino acids 384–1040) and cRAG2 (amino acids 1–383) were co-expressed and purified from 293T cells as described previously^11^,^19^,^21^,^43^. In brief, 293T cells were transfected with Maltose binding protein (MBP) tagged cRAG1/cRAG2 by calcium phosphate method. Cells were harvested after 48 h of transfection and proteins were purified by affinity purification using amylose resin column (New England Biolabs). NBD deleted cRAG1 (Δ462 and Δ402) was co-expressed with cRAG2 and purified from 293T cells as described^28^. In the case of Δ462, amino acids 1 to 461 was deleted from N-terminus of RAG1, while up to 401 amino acids was deleted in Δ402.

### Western blotting

For immunoblot analysis, protein samples were resolved on 12% SDS-PAGE. Following electrophoresis, the protein was transferred to PVDF membrane (Millipore, USA), blocked with 5% skimmed milk powder, probed with primary antibody against His tag and biotinylated anti-rabbit secondary antibody^41^,^42^. The blots were developed using chemiluminscent solution (Immobilon ^TM^ western, Millipore, USA) and scanned by gel documentation system (LAS 3000, Fuji, Japan).

### Electrophoretic mobility shift assay (EMSA)

EMSA was carried out as described previously^20^,^21^. In brief, γ-^32^P labelled oligomeric substrates were incubated with NBD in a buffer containing 10 mM Tris-HCl (pH 7.5), 0.1 mg ml^–1^ BSA, 33 mM KCl, 5 mM CaCl_2_, 2 mM DTT and 6% (v/v) glycerol, for 15 min at 30 °C. Depending on the experimental conditions, 50–200 ng of purified NBD was used for the study. In control reactions, only the binding buffer was used. In case of binding studies with cRAGs, the cRAG1 and cRAG2 complex was incubated with DNA substrates in binding buffer containing 22.5 mM MOPS-KOH (pH 7.0), 20% dimethyl sulfoxide (DMSO), 2.2 mM DTT, 50 mM potassium glutamate, 2% (v/v) glycerol, 100 ng of bovine serum albumin (BSA) per ml at 25 °C for 2 h. The DNA-protein complexes were then separated immediately on 4% native polyacrylamide gel. The gel was dried, signals were detected using a PhosphorImager [FLA9000 (Fuji, Japan)] and products were quantified with Multigauge software. Each experiment described in the present study was done a minimum of three independent times with complete agreement.

### Bio-layer interferometry (BLI)

ForteBio Octet RED 96 (Forte Bio, USA) and streptavidin-coated (SA) sensors (Forte Bio, USA) were used for studying the interactions between NBD and poly T DNA or 12RSS^29^. 1X PBS was used as assay buffer for BLI and the study was conducted at 30 °C. SA sensor tips were hydrated in buffer for 30 min prior to use. The wells in 96-microwell plate were filled with 200 μl of either buffer or sample in each case and agitated at 1200 rpm. The hydrated sensors were immobilized and saturated with biotinylated DNA, which included establishment of a stable baseline with buffer (6 min), loading of sensors with biotinylated DNA 1 μM (6 min) and blocking of excess reactive sites using biocytin (0.04 mg/ml). A reference sensor without biotinylated DNA subjected to the same procedure as the sensors loaded with biotinylated nucleic acids served as a background control. Following immobilization with biotinylated poly T DNA, binding interaction with different concentrations of NBD was carried out, which included baseline (10 min), association (10 min), dissociation (10 min), baseline (10 min) and the Kd value was calculated using curve fit (1:1) model.

### RAG cleavage assay

RAG cleavage assays were carried out in a buffer containing 25 mM MOPS (pH 7.0), 30 mM KCl, 30 mM potassium glutamate and 5 mM MgCl_2_^16^,^19^. Radiolabelled oligomeric DNA substrate and RAG proteins were mixed together and incubated for 1 h at 37 °C. In the control, RAG reaction buffer alone was used. Reactions were terminated by adding loading dye containing formamide, heated for 10 min at 90 °C, cooled on ice, resolved on 15% denaturing polyacrylamide gels and signals were detected using phosphorImager, FLA9000 (Fuji, Japan).

### Reconstitution assay

NBD deleted cRAG1 (Δ462 and Δ402) and cRAG2 were co-expressed and purified from human kidney epithelial cells (HEK 293T) as described earlier^28^. For reconstitution assay, NBD deleted cRAG1 and cRAG2 (Δ462cRAG1/cRAG2 or Δ402cRAG1/cRAG2) were incubated with radiolabelled double-stranded oligomer containing 12RSS in RAG cleavage buffer containing containing 25 mM MOPS (pH 7.0), 30 mM KCl, 30 mM potassium glutamate and 5 mM MgCl_2_, along with purified NBD for 1 h at 37 °C. 12RSS incubated with wild type cRAG1/cRAG2 served as the positive control. Reactions were terminated by adding loading dye containing formamide, denatured for 10 min at 90 °C, resolved on 15% denaturing polyacrylamide gels and visualized with the help of a phosphorImager.

## Additional Information

**How to cite this article**: Raveendran, D. and Raghavan, S. C. Biochemical Characterization of Nonamer Binding Domain of RAG1 Reveals its Thymine Preference with Respect to Length and Position. *Sci. Rep.* **6**, 19091; doi: 10.1038/srep19091 (2016).

## Supplementary Material

Supplementary Information
