# Supplementary material for: Biochemical Characterization of Nonamer Binding Domain of RAG1 Reveals its Thymine Preference with Respect to Length and Position
Source: Sci Rep. 2016 Jan 8;6:19091. doi: 10.1038/srep19091 (PMC4705477; doi:10.1038/srep19091)
Supplement: Supplementary Information [file srep19091-s1.pdf]

Biochemical Characterization of Nonamer Binding Domain of RAG1 Reveals its Thymine Preference with Respect to Length and Position

Deepthi Raveendran and Sathees C. Raghavan*

Department of Biochemistry, Indian Institute of Science, Bangalore, 560 012, India

Supplementary Figure Legends

Table S1. Oligomeric DNA used in the study.

Figure S1. A. Polyacrylamide gel profile showing specificity of NBD binding to 12 RSS. NBD was incubated with radiolabeled 12RSS in the absence (lane 2) or in presence of increasing concentration of unlabelled 12RSS (lanes 3-7; 2, 5, 10, 20, 30 nM). Following incubation (15 min, 30°C) in binding buffer, the reactions were resolved on a 4% native polyacrylamide gel. Lane 1 is oligomeric DNA substrate alone. **B.** Bar diagram showing quantification of NBD binding in presence of increasing concentration of cold 12RSS. **C.** NBD was incubated with labelled 12RSS in the absence (lane 2) or in presence of increasing concentration of unlabelled nonspecific DNA (lanes 3-7; 2, 5, 10, 20, 30 nM). Lane 1 is oligomeric DNA substrate alone. **D.** Bar diagram showing quantification of NBD binding, in presence of increasing concentration of cold nonspecific DNA.

Figure S2. A. Evaluation of NBD binding preference to a heteroduplex DNA containing nonamer of V(D)J recombination. Diagrammatic representation of oligomeric DNA substrates containing heteroduplex DNA harboring nonamer (VII) and scrambled nonamer, highlighted in red (XXXVII). **B.** Polyacrylamide gel profile showing NBD binding to heteroduplex DNA with nonamer and scrambled nonamer at increasing NBD concentrations (50, 100, 150, 200 ng) of protein. The bands due to NBD binding are indicated.

Figure S3. Binding of cRAGs to single and double-stranded DNA with different homopolymeric nucleotides. A. Diagrammatic representation of oligomeric DNA substrates used for the study. **B.** Polyacrylamide gel profile showing cRAG binding to different ss DNA containing poly A, T, G, C and ds DNA with poly A/T and G/C nucleotides. The bands due to NBD binding are indicated. The zoom in image represents the binding at the highlighted rectangle portion following higher exposure of the original gel image. Note the intermolecular G-quadruplex formation in oligomer XI due to presence of K^{+} ions in the reaction buffer.

Figure S4. Bar diagram showing quantification of NBD binding when incubated with different ssDNA containing thymine stretches of different length. For other details refer Figure 6 legend.

Table 1. The sequences of the oligomers used in the study.

Name	Oligomer sequence
AKN1	5′-GATCAGCTGATAGCTACCACAGTGCTACAGACTGGAACAAAAACCCTGCT-3′
AKN2	5′-TAGCAGGGTTTTTGTTCCAGTCTGTAGCACTGTGGTAGCTATCAGCTGAT-3′
AKN3	5′-GATCAGCTGACAGTAGCACAGTGGTAGTACTCCACTCTCTGGCTGTACAAAAACCCTGCT-3′
AKN4	5′-TAGCAGGGTTTTTGTACAGCCAGAGAGTGGAGTACTACCACTGTGCTACTGTCAGCTGAT-3′
AKN11	5′-GACCTGAGGGCGAGCTTTTTTCGAGTAACTTAACAG-3′
AKN18	5′-CTGTTAAGTTACTCGAAAAAAGCTCGCCCTCAGGTC-3′
AKN20	5′-CTGTTAAGTTACTCGCCCCCCGCTCGCCCTCAGGTC-3′
AKN45	5′-GACCTGAGGGCGAGCAAAAAACGAGTAACTTAACAG-3′
AKN46	5′-GACCTGAGGGCGAGCCCCCCCCGAGTAACTTAACAG-3′
AKN47	5′-GACCTGAGGGCGAGCGGGGGGCGAGTAACTTAACAG-3′
AKN48	5′-CTGTTAAGTTACTCGGGGGGGGCTCGCCCTCAGGTC-3′
AKN139	5′-CGCGGATCCGTGCATATCAATAAAGG-3′
AKN150	5′-CGCGGATCCGTGCATATCAATAAAGG-3′
NM32	5′-GACCTGAGGGCGAGCTTTTTTCGAGTAACTTAAACAAAAACCCTGCT-3′
NM33	5′-AGCAGGGTTTTTGTTTAAGTTACTCGTTTTTTGCTCGCCCTCAGGTC-3′
MN37	5′-CCTCTCCCCCTCCCCTCCCCTCCCTCGCGC-3′
MN38	5′-GCGCGAGGGAGGGGAGGGGAGGGGGAGAGG-3′
MN45	5′-AGAAGGGGGAGGGGAGGGAGAGAGGGGGCGCCG-3′
MN46	5′-CGGCGCCCCCTCTCTCCCTCCCCTCCCCCTTCT-3′
DR3	5′-AAGGAAAAAAGTCGACTTACTCATAGCGCTGCAG-3′
SCR252	5′-CTGTTAAGTTACTCGTTTTTTGCTCGCCCTCAGGTC-3′
MS3	5´-TTTTTTTTTTTTTTTTTTTTTTTTTTTTTTTTTTT-3´
MS5	5´-AAAAAAAAAAAAAAAAAAAAAAAAAAAAAAAAAAA-3
MS9	5´- GGGGGGGGGGGGGGGGGGGGGGGGGGGGGGGGGG-3´
MS4	5´-CCCCCCCCCCCCCCCCCCCCCCCCCCCCCCCCCCC-3´
MS6	5´-TTTTTTTTTTTTTTTTTTTTTTTTTTTTTTTTTTTTTTTTTTTTTTTTTTTTTTTTTTTTTTTTTTTTTTTTTT-3´
SCR52	5´-AAAAAAAAAAAAAAAAAAAAAAAAAAAAAAAAAAAAAAAAAAAAAAAAAAAAAAAAAAAAAAAAAAAAAAAAAA-3´
MS103	5′-TTTTTTTTTT-3′
MS102	5′-TTTTTTTTTTTTTTTT-3′
MS100	5′-TTTTTTTTTTTTTTTTTTTTTT-3′
MS101	5′-TTTTTTTTTTTTTTTTTTTTTTTTTTTT-3′
DR38	5′-AAAAAAAAAA-3′
DR39	5′-AAAAAAAAAAAAAAAA-3′
DR40	5′-AAAAAAAAAAAAAAAAAAAAAA-3′
DR41	5′-AAAAAAAAAAAAAAAAAAAAAAAAAAAA-3′
MS12	5´-GAGTCTGATCTTACCTCTTAACTTCGCTACTTTTT-3´
MS14	5´-GAGTCTGATCTTACCTCTTAACTTCTTTTTTTTTT-3´
MS16	5´-GAGTCTGATCTTACCTCTTATTTTTTTTTTTTTTT-3´
MS18	5´-GAGTCTGATCTTACCTTTTTTTTTTTTTTTTTTTT-3´
MS22	5´-TTTTTTTTTTGAGTCGGATCTTACGTCGTAACTTCGCTAC-3´
MS25	5´-GAGACTGATCTTACCTCTTAACTACTTTTTGACAC-3´
DR59	5′-GACCTGAGGGCGAGCTTTTTTCGAGTAACTTAAGCGGAGGTCCTGCT-3′
DR60	5′-AGCAGGACCTCCGCTTAAGTTACTCGTTTTTTGCTCGCCCTCAGGTC-3′
DR61	5′-GAGTCTGATCTTACCTCTTAACTTCGCTACCTTTT-3′
DR62	5′-GAGTCTGATCTTACCTCTTAACTTCGCTACCATTT-3′

Table S1

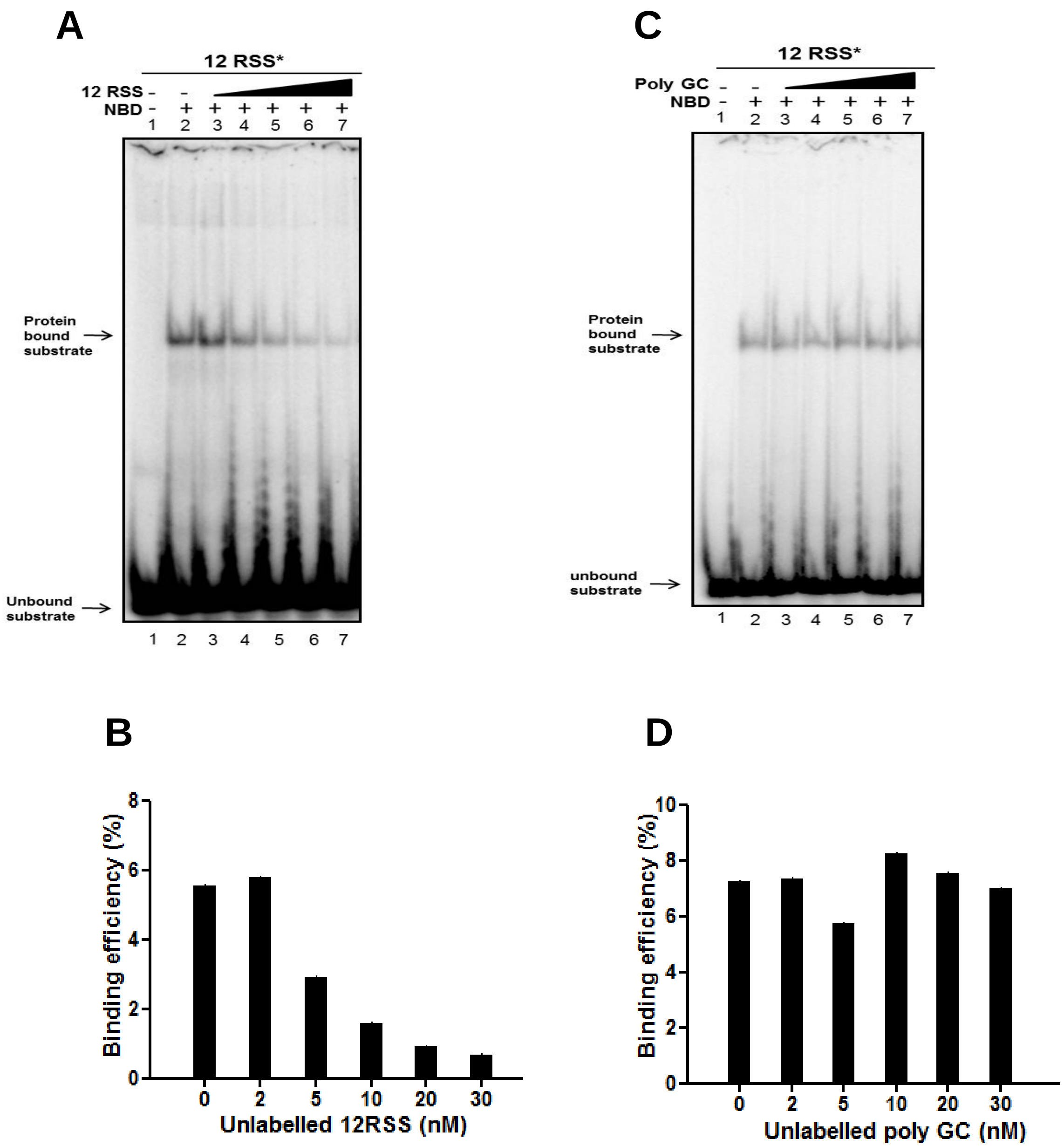

A
12 RSS*
12 RSS - -
NBD - + + + + + +
1 2 3 4 5 6 7
Protein bound substrate
Unbound substrate
1 2 3 4 5 6 7
C
12 RSS*
Poly GC - -
NBD - + + + + + +
1 2 3 4 5 6 7
Protein bound substrate
unbound substrate
1 2 3 4 5 6 7
B
Binding efficiency (%)
0 2 4 6 8
0 2 5 10 20 30
Unlabelled 12RSS (nM)
D
Binding efficiency (%)
0 2 4 6 8 10
0 2 5 10 20 30
Unlabelled poly GC (nM)

Figure S1

A

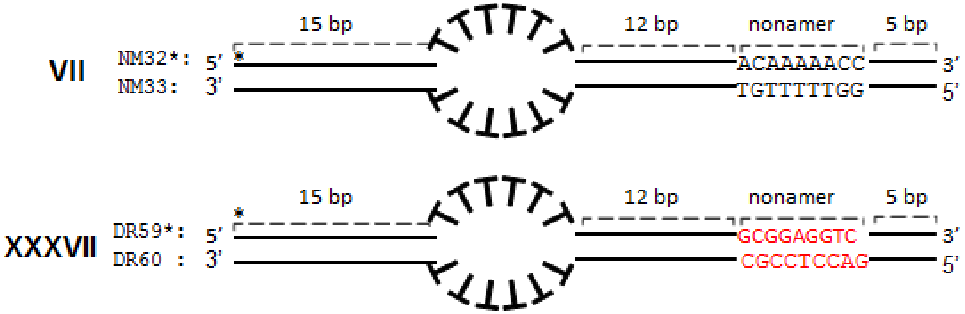
15 bp
12 bp
nonamer
5 bp
VII
NM32*: 5'
NM33: 3'
ACAAAAACC
TGTTTTTGG
3'
5'
XXXVII
DR59*: 5'
DR60 : 3'
GCGGAGGTC
CGCCTCCAG
3'
5'

B

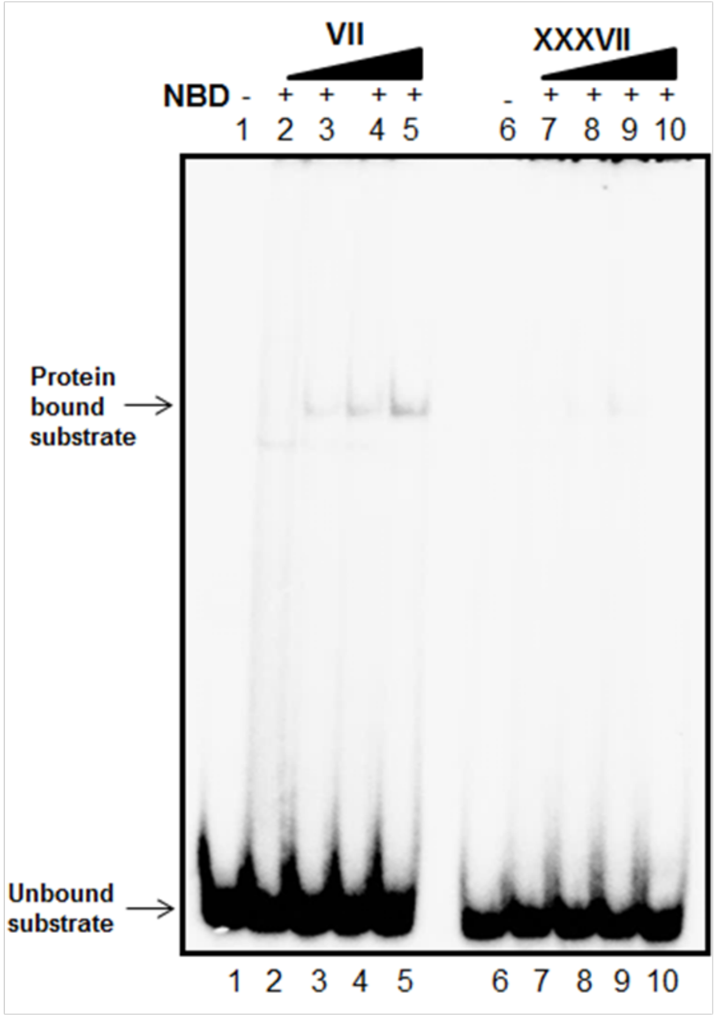
VII
XXXVII
NBD - + + + + - + + + +
1 2 3 4 5 6 7 8 9 10
Protein bound substrate
Unbound substrate
1 2 3 4 5 6 7 8 9 10

Figure S2

A

**IX**	MS5*:	5′-A*AAAAAAAAAAAAAAAAAAAAAAAAAAAAAAAAAA-3′
**X**	MS3*:	5′-T*TTTTTTTTTTTTTTTTTTTTTTTTTTTTTTTTTT-3′
**XI**	MS9*:	5′-G*GGGGGGGGGGGGGGGGGGGGGGGGGGGGGGGGGG-3′
**XII**	MS4*:	5′-C*CCCCCCCCCCCCCCCCCCCCCCCCCCCCCCCCCC-3′
**XIII**	MS5*:	5′-A*AAAAAAAAAAAAAAAAAAAAAAAAAAAAAAAAAA-3′
	MS3:	3′-TTTTTTTTTTTTTTTTTTTTTTTTTTTTTTTTTTT-5′
**XIV**	MS9*:	5′-G*GGGGGGGGGGGGGGGGGGGGGGGGGGGGGGGGGG-3′
	MS4:	3′-CCCCCCCCCCCCCCCCCCCCCCCCCCCCCCCCCCC-5′
**I**	AKN1*:	5′-GATCAGCTGATAGCTAC CACAGTGCTACAGACTGGAACAAAAACC CTGCT-3′
	AKN2:	3′- TAGTCGACTATCGATG GTGTCACGATGTCTGACCTTGTTTTTGG GACGAT-5′

B

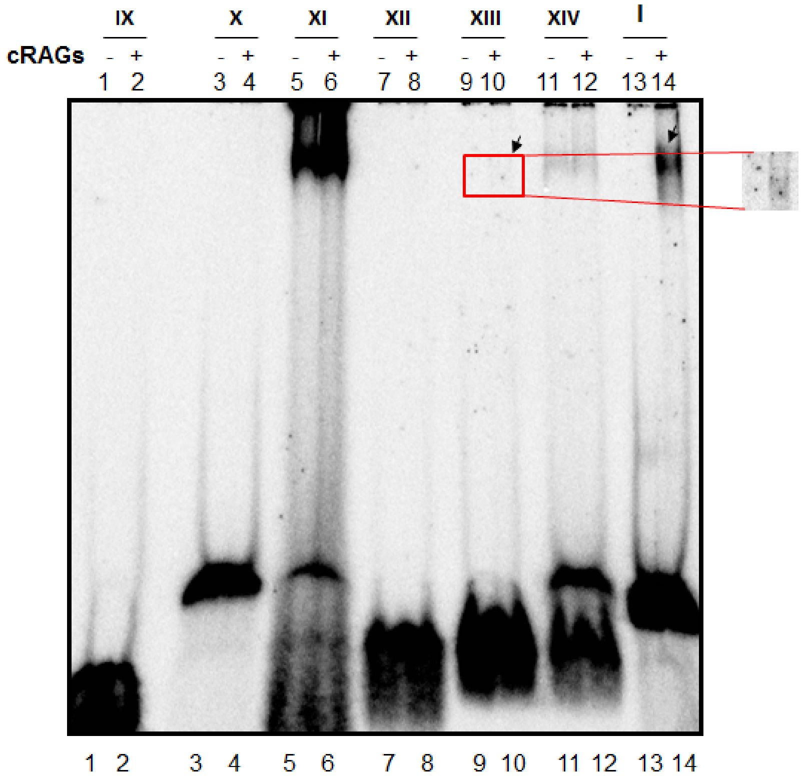

Figure S3

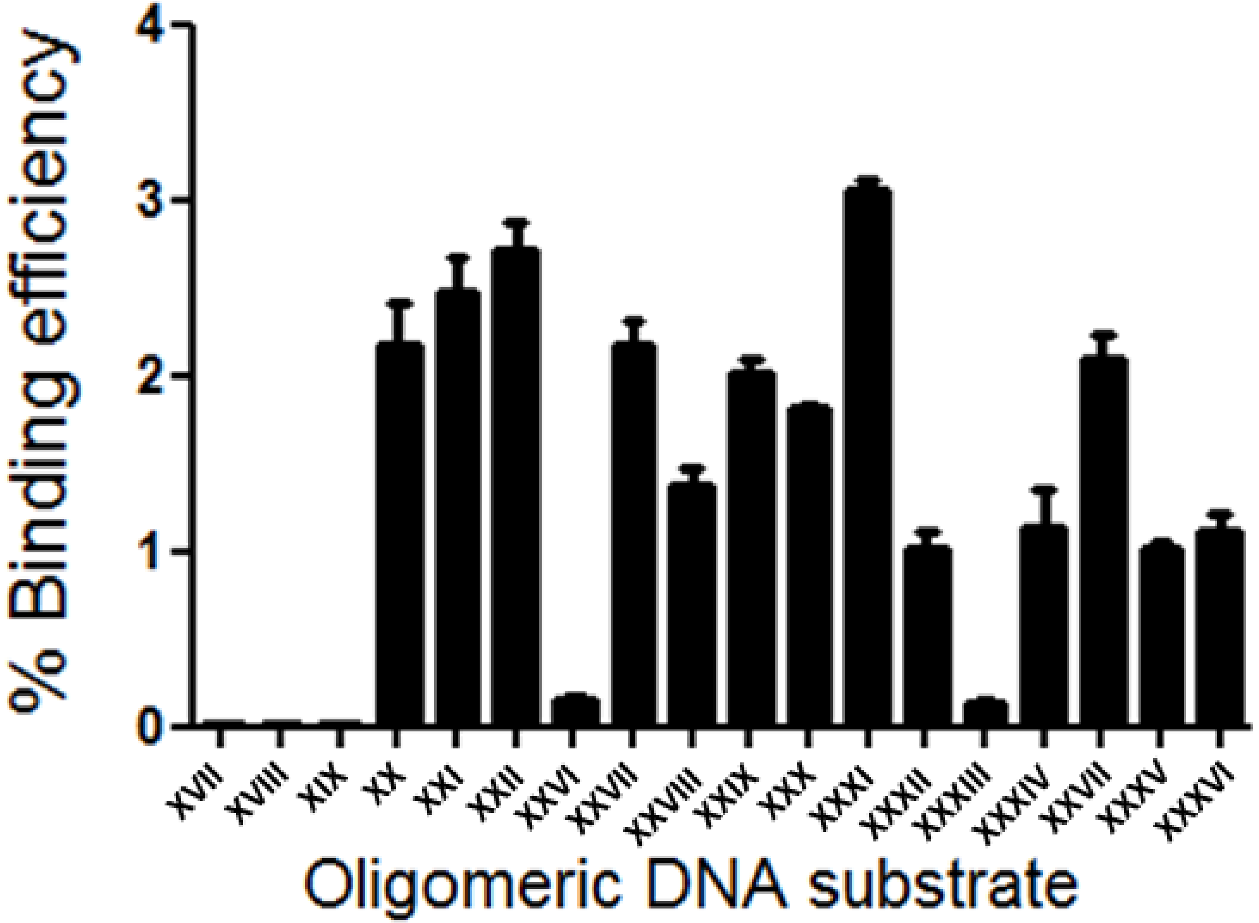

% Binding efficiency
4
3
2
1
0
XVII
XVIII
XIX
XX
XXI
XXII
XXVI
XXVII
XXVIII
XXIX
XXX
XXXI
XXXII
XXXIII
XXXIV
XXVII
XXXV
XXXVI
Oligomeric DNA substrate

Figure S4
